# Midlife development of type 2 diabetes and hypertension in women by history of hypertensive disorders of pregnancy

**DOI:** 10.1186/s12933-018-0764-2

**Published:** 2018-09-10

**Authors:** Simon Timpka, Amanda Markovitz, Tommy Schyman, Ingrid Mogren, Abigail Fraser, Paul W. Franks, Janet W. Rich-Edwards

**Affiliations:** 1 Connors Center for Women’s Health and Gender Biology, Brigham and Women’s Hospital and Harvard Medical School, Boston, MA USA; 2 Genetic and Molecular Epidemiology Unit, Lund University Diabetes Centre, Clinical Sciences Malmö, Lund University, Malmö, Sweden; 3 Harvard T.H. Chan School of Public Health, Boston, MA USA; 4 Forum South, Clinical Studies Sweden, Skåne University Hospital, Lund, Sweden; 5 Department of Clinical Sciences, Obstetrics and Gynecology, Umeå University, Umeå, Sweden; 6 Population Health Sciences, Bristol Medical School, University of Bristol, Bristol, UK; 7 MRC Integrative Epidemiology Unit at the University of Bristol, University of Bristol, Bristol, UK; 8 NIHR Biomedical Research Centre, The University Hospitals Bristol NHS Foundation Trust and the University of Bristol, Bristol, UK; 9 Department of Public Health and Clinical Medicine, Umeå University, Umeå, Sweden

**Keywords:** Hypertensive disorders of pregnancy, Preeclampsia, Gestational hypertension, Epidemiology, Type 2 diabetes, Hypertension

## Abstract

**Background:**

Women with history of hypertensive disorders of pregnancy (HDP) are at increased risk of early onset cardiovascular disease and type 2 diabetes (T2D). We aimed to investigate the extent to which HDP is also associated with midlife development of T2D and hypertension above and beyond established risk factors.

**Methods:**

We included parous women who attended population-based structured clinical visits at age 50 and 60 years in Sweden 1991–2013 (N = 6587). Women with prior diabetes mellitus, stroke, or ischemic heart disease at age 50 years were excluded. Data on reproductive history were collected from registries. To study the association between history of HDP and the between-visits development of T2D, hypertension, and clinical risk factors of cardiometabolic disease (body mass index (BMI), blood pressure, and total cholesterol), we utilized multivariable adjusted regression models (logistic, log binomial, and linear regression, respectively). Models included data on outcome risk factors at age 50 years, e.g. BMI, 75 g 2 h oral glucose tolerance test result, and mean arterial pressure, respectively.

**Results:**

Between ages 50 and 60 years, 5.8% of initially disease-free women developed T2D and 31.6% developed hypertension. History of HDP was associated with increased risk of developing T2D between age 50 and 60 years even when adjusting for risk factors, including BMI, at age 50 years (odds ratio (OR) 1.96, 95% confidence interval (CI) 1.29–2.98). By contrast, the higher risk of developing hypertension observed in women with history of HDP (relative risk (RR) 1.47, 95% CI 1.22–1.78) was attenuated when adjusted for risk factors (RR 1.09, 95% CI 0.94–1.25). Participants with a history of HDP had higher mean BMI and blood pressure at age 50 years, with levels roughly corresponding to those observed at age 60 years in unaffected women.

**Conclusions:**

Women with history of HDP are not only at higher risk of cardiometabolic disease during their reproductive years, but HDP is also associated with midlife T2D development above and beyond established risk factors.

**Electronic supplementary material:**

The online version of this article (10.1186/s12933-018-0764-2) contains supplementary material, which is available to authorized users.

## Background

Hypertensive disorders of pregnancy (HDP: preeclampsia and gestational hypertension) are potentially life-threatening obstetric complications that affect 3–10% of all pregnancies [1]. Women with a history of HDP also develop hypertension at a younger age, [2–4] and are at twice the risk of developing type 2 diabetes (T2D) [5, 6] and cardiovascular disease (CVD) post-pregnancy [7–10]. This has led to calls for HDP history to be used for identifying women with an adverse risk factor profile at a young age [11, 12]. However, less is known about the clinical relevance of information on HDP history for cardiometabolic disease prevention in middle age, especially when concomitantly considered with cardiometabolic risk factors.

In this study we were interested in the association between HDP history and the development of T2D and hypertension in a cohort of women with clinical visits at age 50 and 60 years. To mimic a primary prevention setting, we sought to estimate associations independently of any cardiometabolic deterioration already clinically apparent at age 50 years, and included predictors of later cardiometabolic health in our models. To further understand the clinical relevance of the association between history of HDP and cardiometabolic risk factors [including blood pressure and body mass index (BMI)] in midlife, we also investigated these associations at age 50 years, the respective change in any association by age 60 years, and compared the associations with those attributable to the 10 years of older age between visits. Our primary hypothesis was that in women initially free of severe cardiovascular disease, history of HDP is positively associated with developing T2D and hypertension between age 50 and 60 years also when accounting for conventional predictors of cardiometabolic deterioration measurable at age 50 years.

## Methods

In this prospective cohort study, we utilized a combination of clinical data, collected in a standardized population-based setting in northern Sweden, and comprehensive registry resources on reproductive history including pregnancy complications. Data on hospitalizations and cause of death were collected from the Swedish in-patient and Cause of Death Registry, respectively. Data on place of residence and educational level were collected from Statistics Sweden (Örebro, Sweden).

### Study sample identification

**Fig. 1:**
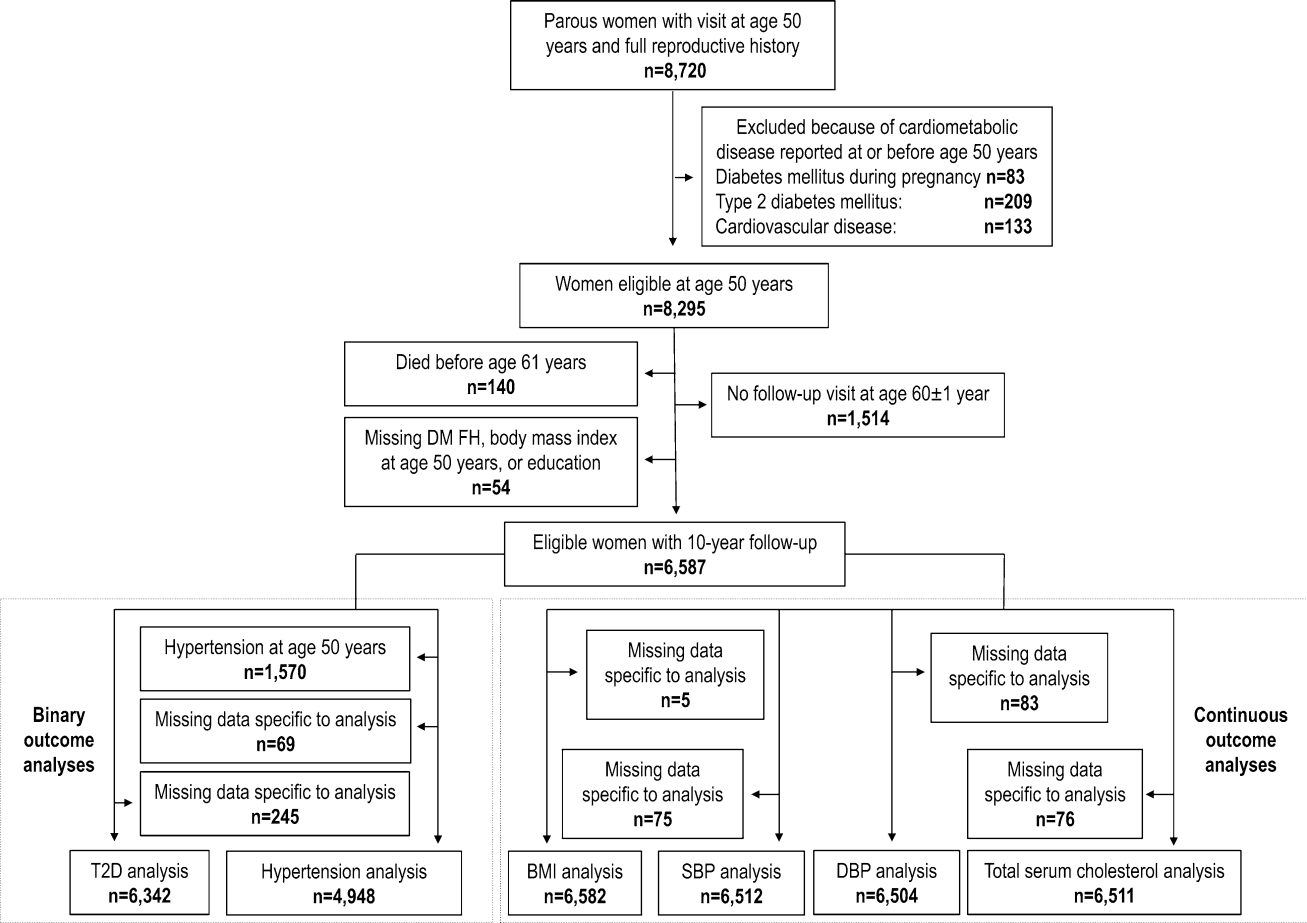
Identification of study sample. The figure shows the identification of each analytic sample used to examine the associations between history of HDP and 10 year risk of T2D and hypertension in women 50 years of age. *BMI* body mass index, *DBP* diastolic blood pressure, *DM FH* diabetes mellitus family history, *SBP* systolic blood pressure, *T2D* type 2 diabetes

We included parous women who attended population-based structured clinical visits in primary care (The Västerbotten Intervention Program) at age 50, and again 10 years later, both visits occurring between 1991 and 2013 in northern Sweden (Fig. 1). Of 11,244 potentially eligible parous women, we included 8720 women with full reproductive history. This was defined as living in the geographical area covered by the regional pregnancy register in 1968, when data on place of residence for all participants were available. Women with known diabetes mellitus during pregnancy were excluded as these women are already known to have a very high risk of T2D and because we did not have data on pre-pregnancy diabetes status. The identification of the study sample is shown in Fig. 1. We excluded women with severe cardiovascular disease before the first visit at age 50 years (stroke, transient ischemic attack, myocardial infarction, or angina) according to in-patient ICD codes (version 9 or 10) or self-report, including anti-angina medication. As there was a small proportion of missing data in our final sample (< 5% missing any variable), we performed complete case analyses for each outcome.

### Data on reproductive history

Data on reproductive history, including HDP (hypertension in pregnancy, preeclampsia, and eclampsia), were collected from regional to national delivery registries. In short, we utilized a local birth registry for the period 1955–1972 [13] (see Additional file 1) and the national Swedish Medical Birth Registry for the period from 1973 onwards. We utilized ICD codes to identify women with a reported diagnosis of HDP or diabetes mellitus during pregnancy (see Additional file 1 for ICD codes).

### Cardiometabolic measurements at age 50 years visit and at the 10-year follow-up

All women aged 50 and 60 years living in the county of Västerbotten in northern Sweden have since 1991 been invited to undergo standardized health assessments, focused on cardiometabolic health prevention, at their primary care centers [14, 15]. Study visits are conducted by a nurse and, for participants who are not already diagnosed with diabetes mellitus, typically include a 75 g 2 h oral glucose tolerance test (OGTT). For the purpose of this study, diabetes mellitus was defined either as (I) self-report of previous diagnosis (II) fasting capillary plasma glucose (≥ 7.0 mmol/l) (III) 2 h capillary plasma glucose > 12.1 (IV) currently taking anti-diabetic drugs or (V) reporting lifestyle treatment for diabetes. Total serum cholesterol was analyzed locally at each primary health care center using a Reflotron bench analyzer until August 2009 and then onwards at the central clinical chemistry lab at Umeå University Hospital. From 1991 to August 2009, blood pressure was measured with a sphygmomanometer with the participants in a horizontal position after a five min rest. From September 2009 onwards, blood pressure was measured with the participants in a seated position. As the large majority of the blood pressure measurements in this study was performed prior to 2009, we converted all measurements taken with the participant in a seated position to horizontal values by using age-specific formulae [16]. We defined hypertension as either (I) systolic blood pressure (SBP) ≥ 140 mmHg (II) diastolic blood pressure (DBP) ≥ 90 mmHg or (III) self-report use of anti-hypertensive drugs. Mean arterial pressure was calculated as DBP + [(SBP − DBP)/3]. Participant body weight and height were measured by a nurse and BMI was calculated as (weight in kg)/(height in m)^2^. Smoking habits, family history of diabetes mellitus, and family history of CVD were collected via self-administered questionnaires.

As previously recommended, [17] for these analyses we added a constant to the clinical measurement to compensate for anti-hypertensive or lipid-lowering medication, respectively. When analyzing continuous blood pressure, we added a standard 15 mmHg to SBP and 10 mmHg to DBP if participants reported anti-hypertensive drug use [17]. When analyzing continuous serum cholesterol, we added 1.336 mmol/l for participants who reported use of lipid lowering drugs [18].

### Statistical analyses

In the analyses of binary outcomes, we investigated the associations between history of HDP and 10-year risk of developing of T2D and hypertension between 50 and 60 years of age. We utilized logistic regression for T2D and, as we anticipated a high prevalence of hypertension at follow-up, we utilized log binomial regression to directly obtain a risk ratio and 95% confidence interval (CI) for hypertension. In Model I, we included HDP history. In Model II, we adjusted for family history of CVD and T2D, BMI, smoking, education level, and year of birth. In the main model (Model III), we further included a measure of already accumulated risk of the outcome at age 50 years (capillary glucose at 2 h post OGTT for T2D and mean arterial pressure at age 50 years for the hypertension analyses, respectively).

In the analyses of continuous outcomes, we investigated the association between history of HDP and several cardiometabolic risk factors (BMI, SBP, DBP, log total serum cholesterol) at ages 50 and 60 years and whether these associations changed with age. To do so we used linear mixed models with fixed effects for the between-participant variation. We estimated 95% CIs and calculated complementary p-values. In Model I, we included HDP history, age at clinical visit, and an interaction term between the HDP history and age. In Model II, we adjusted for family history of CVD and family history of diabetes mellitus. In the main model (Model III), we further included education level, smoking, year of birth, BMI (when BMI was not the dependent variable), as well as interaction terms for age and smoking and age and BMI (when BMI was not the dependent variable). Participants eligible for these analyses had no missing data at either of their visits, resulting in 6504–6582 participants contributing data (Fig. 1).

As an additional analysis we wanted to replicate previous findings on the association between history of HDP and post-pregnancy development of T2D and hypertension. To do so, we used regression models, as described above for T2D and hypertension, but did not exclude women based on their cardiometabolic status at age 50 years. Instead, we utilized the data from the initial age 50 years clinical visit as outcome data. In addition to a crude analysis, we analyzed models adjusted for education level and family history. All analyses were performed using SAS 9.4.

## Results

Table 1 shows the participant characteristics stratified by history of HDP. In total, 285 (4.3%) women had at least one pregnancy complicated by HDP and resulting in a birth. Whereas women with a history of HDP had similar educational level as women not affected, they had markedly higher blood pressure at ages 50 and 60 years. During the 10 years of follow-up from age 50 years, 385 (5.8%) participants developed T2D of whom 36 women (9.4%) had a history of HDP. Of the 4958 normotensive participants at age 50 years, including 10 participants with missing data and not included in the regression model, 1568 (31.6%) had developed hypertension 10 years later.

**Table 1:** Study sample characteristics by history of HDP

	No history of HDP		HDP history	
		Missing data, N		Missing data, N
N participants (%)	6302 (95.7)	–	285 (4.3)	–
Age at age 50 visit, years mean ± SD	50.1 ± 0.3	–	50.1 ± 0.3	–
Age at age 60 visit, years mean ± SD	60.0 ± 0.2	–	60.0 ± 0.2	–
Family history of CVD, N (%)	1787 (28.4)	–	92 (32.3)	–
Family history of diabetes, N (%)	1968 (31.2)	–	99 (34.7)	–
MAP at 50 years, mmHg mean ± SD	95 ± 13	68	106 ± 14	4
SBP at 50 years, mmHg mean ± SD	127 ± 19	61	141 ± 20	4
SBP at 60 years, mmHg mean ± SD	136 ± 20	5	146 ± 19	–
DBP at 50 years, mmHg mean ± SD	79 ± 11	67	88 ± 13	4
DBP at 60 years, mmHg mean ± SD	82 ± 11	7	87 ± 11	–
BMI at 50 years, kg/m^2^ mean ± SD	25.3 ± 4.0	–	26.5 ± 4.5	–
BMI at 60 years, kg/m^2^ mean ± SD	26.4 ± 4.5	5	27.4 ± 4.8	–
2 h glucose post-OGTT at age 50 years, mmol/l mean ± SD	6.78 ± 1.3	233	6.92 ± 1.4	11
Education level, N (%)		–		–
Elementary school or less	1167 (18.5)		56 (19.7)*	
High school	3390 (53.8)		157 (55.1)*	
College/University	1745 (27.7)		72 (25.3)*	
Cholesterol age 50 years, mmol/l median (IQR)	5.62 (4.99; 6.35)	59	5.83 (5.10; 6.65)	1
Cholesterol age 60 years, mmol/l median (IQR)	5.95 (5.30; 6.64)	11	6.00 (5.38; 6.76)	1
Smoking age 50 years, N (%)	1449 (23.0)	–	54 (19.0)	–
Smoking age 60 years, N (%)	911 (14.5)	–	32 (11.2)	–

Dashes in columns are markers of no missing data in the study sample

*BMI* body mass index, *DBP* diastolic blood pressure, *MAP* mean arterial blood pressure, *OGTT* oral glucose tolerance test, *SBP* systolic blood pressure

* Percentage not adding up to 100% due to rounding

### Confirmation of the association between history of hypertensive disorders of pregnancy and cardiometabolic disease at age 50 years

The analyses focusing on cardiometabolic disease development between pregnancy and age 50 years included 8601 women in the T2D analysis and 8476 women in the hypertension analysis. In these analyses, history of HDP was associated with an increased odds ratio (OR) of T2D in both the crude (OR 3.26, 95% CI 2.13–4.99) and adjusted analysis (OR 3.13, 95% CI 2.04–4.80). Likewise, HDP history in any pregnancy was associated with an increased risk of hypertension at age 50 years (relative risk (RR) 2.27, 95% CI 2.06–2.51 and 2.22, 95% CI 2.01–2.44, respectively).

### History of hypertension disorders of pregnancy and midlife development of cardiometabolic disease

Table 2 shows history of HDP to be associated with incident T2D between age 50 and 60 years, even after adjusting for cardiometabolic health, including glucose response to OGTT, at age 50 years (OR 1.96, 95% CI 1.29–2.98). History of HDP was associated with incident hypertension between age 50 and 60 years (crude RR 1.47, 95% CI 1.22–1.78) but this association was abrogated in the model that adjusted for blood pressure and other risk factors at age 50 years (RR 1.09, 95% CI 0.94–1.26).

**Table 2:** HDP and incident type 2 diabetes and hypertension between age 50 and 60 years

Model	OR of T2D (95% CI)^a^	RR of hypertension (95% CI)^b^
Model I	2.35 (1.61; 3.44)	1.47 (1.22; 1.78)
Model ÏI	1.98 (1.33; 2.95)	1.30 (1.10; 1.53)
Model III	1.96 (1.29; 2.98)	1.09 (0.94; 1.25)

Model I includes: history of HDP (yes or no)

Model II additionally includes: family history of diabetes mellitus (yes or no), family history of cardiovascular disease (yes or no), body mass index at age 50 years, education level (9 years or less, 10–12 years, > 12 years), smoking status at age 50 years (yes or no), and year of birth

Model III additionally includes: capillary glucose at 2 h following 75 g OGTT at age 50 years (for T2D) or mean arterial pressure at age 50 years (for hypertension)

*CI* confidence interval, *HDP* hypertensive disorders of pregnancy, *OR* odds ratio, *RR* relative risk, *T2D* type 2 diabetes mellitus, *OGTT* oral glucose tolerance test

^a^Modelled with logistic regression

^b^Modelled with log binomial regression

### History of hypertensive disorders of pregnancy and cardiometabolic risk factor status at age 50 and 60 years

Table 3 shows history of HDP to be associated with approximately one unit higher BMI at both age 50 and 60 years, as evidenced in the second column and the lack of interaction between age and HDP history in the sixth column. History of HDP was also associated with higher SBP and DBP and this difference at age 50 years (on the order of 12 mmHg SBP and 8 mmHg DBP) are greater or comparable to the age-related difference (roughly 10 mmHg SBP and 4 mmHg DBP) between visits at age 50 and 60 years in all women. However, the absolute effect size attributable to history of HDP was roughly 3 mmHg lower at age 60 years for both DBP and SBP compared to age 50 years (p for interaction < 0.05 for all BP comparisons). Although history of HDP was associated with higher total serum cholesterol at both age 50 and 60 years, these associations were small compared to the age-related increase.

**Table 3:** The association of HDP history and CVD risk factors at age 50, the change in CVD risk factors from age 50 to age 60 years, and the interaction between HDP history and age

	Age 50, no HDP history	Mean difference^a^ at age 50 years in women with history of HDP (95% CI)	p, history of HDP	Mean difference, age 60 vs. 50 years (95% CI)^b^	p, age	Interaction, history of HDP and age 60 years (95% CI)^c^	p, interaction
BMI, kg/m^2^							
Model I	Reference	1.16 (0.66; 1.66)	< 0.001	1.12 (1.06; 1.17)	< 0.001	− 0.15 (− 0.42; 0.12)	0.27
Model II	Reference	1.13 (0.63; 1.63)	< 0.001	1.12 (1.06; 1.17)	< 0.001	− 0.15 (− 0.42; 0.12)	0.27
Model III	Reference	1.09 (0.59; 1.59)	< 0.001	1.06 (1.00; 1.12)	< 0.001	− 0.15 (− 0.42; 0.12)	0.27
SBP, mmHg							
Model I	Reference	13.5 (11.2; 15.8)	< 0.001	8.28 (7.85; 8.72)	< 0.001	−2.84 (− 4.94; − 0.73)	0.01
Model II	Reference	13.3 (11.1; 15.6)	< 0.001	8.28 (7.85; 8.72)	< 0.001	−2.84 (− 4.94; − 0.73)	0.01
Model III	Reference	11.5 (9.32; 13.7)	< 0.001	10.2 (7.44; 12.9)	< 0.001	− 2.41 (− 4.49; − 0.33)	0.02
DBP, mmHg							
Model I	Reference	*8.58* (7.23; 9.93)	< 0.001	2.46 (2.20; 2.73)	< 0.001	−3.15 (− 4.42; − 1.89)	< 0.001
Model II	Reference	8.50 (7.15; 9.85)	< 0.001	2.46 (2.20; 2.73)	< 0.001	− 3.15 (− 4.42; − 1.89)	< 0.001
Model III	Reference	7.55 (6.25; 8.86)	< 0.001	4.06 (2.42; 5.71)	< 0.001	− 2.91 (− 4.18; − 1.65)	< 0.001
Total serum cholesterol, %^a^							
Model I	Reference	3.9 (1.4; 6.4)	0.002	5.1 (4.5; 5.7)	< 0.001	− 1.9 (− 4.7; 1.0)	0.20
Model II	Reference	3.8 (1.3; 6.3)	0.003	5.1 (4.5; 5.7)	< 0.001	− 1.9 (− 4.7; 1.0)	0.20
Model III	Reference	3.5 (1.0; 6.0)	0.007	16.8 (13.1; 20.5)	< 0.001	− 1.5 (− 4.3; 1.4)	0.30

Model I includes: history of HDP (yes or no), age at visit (50 or 60 years), interaction term between HDP and age at visit

Model II additionally includes: family history of diabetes mellitus (yes/no), family history of cardiovascular disease (yes or no)

Model III additionally adjusts for: smoking (yes or no), interaction term between age and smoking, education level (9 years or less, 10–12 years, > 12 years), year of birth, BMI (not in model with BMI as outcome), interaction between age and BMI (not in model with BMI as outcome)

*BMI* body mass index, *CI* confidence interval, *CVD* cardiovascular disease, *DBP* diastolic blood pressure, *HDP* hypertensive disorders of pregnancy, *OGTT* oral glucose tolerance test, *SBP* systolic blood pressure

^a^For cholesterol the results are presented as difference in percentage as the outcome is log transformed

^b^The effect estimate of 10 years age increase from age 50 years is shown for comparison with the effect estimate of HDP history at age 50 years

^c^Column shows the difference in associations with HDP history at age 60 years compared to age 50 years

### Additional analyses

When we compared the characteristics at age 50 years for women who did and did not attend the follow-up visit, there was no difference in educational level or proportion of women with history of HDP (Additional file 1: Table S1). However, women who did not attend the age 60 years visit were more likely to be smokers at age 50 years and had a tendency of worse mean OGTT result or being hypertensive at the initial visit. Women (N = 2524) who were excluded due to missing reproductive history had similar prevalence of diabetes at age 50 years compared to the study sample (Additional file 1: Table S2). The excluded women had higher level of education and slightly lower blood pressure at age 50 years. However, the absolute difference in blood pressure was small compared to the estimate associated with having a history of HDP in the main sample.

## Discussion

We found history of HDP to be positively associated with incident T2D in women between age 50 and 60 years even after adjustment for clinical risk factors at age 50 years, including OGTT result. By contrast, for hypertension this association was abrogated when we adjusted for risk factors, including MAP. Although the absolute difference in blood pressure was smaller by age 60 years, women with a history of HDP had higher mean BMI and blood pressure at both age 50 and 60 years. Their respective level at age 50 years roughly corresponded to those observed at age 60 years in unaffected women.

### Hypertensive disorders of pregnancy as a predictor of T2D

In our main analysis we have included women without T2D or CVD at age 50 years, controlled for their risk at 50 years, and followed them for 10 years. This approach is novel in the field and, by and large, it mimics a primary prevention setting in which women not yet diagnosed with severe cardiometabolic disease seek advice on prevention and disease risk from their health care provider. To replicate the decision process in the primary care setting, we include known predictors of these outcomes likely to be available to the primary care clinician, to investigate the additional value of HDP history as a clinical predictor above and beyond what is already considered by practitioners. The results suggest that information on history of HDP may be clinically useful for the prediction of midlife T2D development in women. The strong link between HDP history and metabolic function is supported by a previous study in which post-pregnancy obesity modified the association between history of HDP and hypertension [19]. When we investigated the relative risk of disease by HDP history at age 50 years, we in this study also confirm previous results suggesting women with history of HDP have increased risk of T2D and hypertension during the decades following pregnancy [4, 8]. Sattar et al. suggested that pregnancy complications constitute potential susceptibility to future cardiometabolic disease [12] and our results suggest that history of HDP actually has potential as a clinical predictor of T2D in middle age. This susceptibility could potentially be mediated through a more adverse vascular, inflammatory and/or insulin resistant [20] predisposition that is tentatively revealed during pregnancy.

Fraser et al. reported that women with either a history of preeclampsia or gestational hypertension had worse risk factor profile and higher calculated CVD risk at mean age 48 years [21]. In several studies, history of HDP has also been reported to be associated with increased risk of hypertension post-pregnancy [2, 22]. Our results suggest that history of HDP is associated with hypertension development midlife, but that this association is largely attenuated when blood pressure at the beginning of the 10-year interval is accounted for. Drost et al. reported on the longitudinal changes of cardiometabolic risk factors in middle age by HDP history [23]. In their analysis, history of HDP was associated with higher blood pressure but compared to this study the effect estimates were smaller. However, the prevalence of history of HDP in the study was noticeably high at 20%. Our results also supports that women with history of HDP have higher BMI compared to other parous women, [24] but the absolute difference did not increase during midlife.

Several studies suggest an association also between maternal HDP and worse offspring metabolic health later in life [25, 26]. However, the extent to which this association can translate into improved prediction in adulthood above and beyond established determinants is unknown.

### Limitations

By combining population-based clinically collected data with registry resources, we have assembled a large sample with objectively measured outcome data and complete reproductive history. However, the study also has potential limitations. We report a loss to follow-up of 20% at the age 60 years visit. While there was no difference in history of HDP status or education level by having attended the follow-up visit, the group that did not appeared to have a higher proportion of smokers and worse OGTT result. Regardless, our main study results depend on the assumption that loss of follow-up between study visit 1 and 2 does not induce major bias. As we mainly had outcome data registered at the follow-up visits at age 60 years, we did not utilize a survival model but analyzed the outcomes by that time point. We did not have full reproductive history on all women attending the age 50 years visit. As the reproductive data were mainly collected from a comprehensive regional register, it is likely that women missing reproductive history had immigrated to the region during or after their childbearing years. We do not think that it is plausible that the presented associations would vary greatly (i.e. presence of an interaction) between women who have delivered any children outside the counties covered by the register compared to those who have not. Still, additional studies in samples of other populations are needed to confirm the novel results we present. Of note is also that GDM was yet to be established as a distinct diagnostic entity in the 1950–1960s, and we do not have data on pre-pregnancy diagnoses. Thus, we cannot separate GDM from diabetes mellitus present prior to pregnancy. However, women with GDM or diabetes mellitus are excluded from the analytical sample in the main analysis. Similarly, we are not able to exclude women with hypertension prior to pregnancy. The HDP diagnoses are mostly collected from a regional register in which the specific accuracy has not been evaluated. However, data from Norway suggest that diagnoses on HDP set in the 1960s were of reasonable quality and provided some separation even between preeclampsia and gestational hypertension [27].

### Clinical relevance and future studies

In contrast to the guidelines concerning GDM, which currently recommend regular screening for T2D, [28] the guidelines for follow-up after HDP are less specific [29]. Our results show that history of HDP is not only a post-pregnancy risk factor of T2D, but also a predictor of midlife T2D development even after adjusting for post-pregnancy metabolic status. Nonetheless, the clinical utility of history of HDP should also be investigated in less ethnically homogenous populations. As the awareness of the link between HDP history and later cardiometabolic disease is insufficient among physicians in the United States, [30, 31] efforts are needed to inform clinical practice regardless. By conveying the message of the worse cardiometabolic status associated with history of HDP through comparisons with age-related decline, [32] the communication of guidelines and their penetration to clinical practice might be facilitated.

## Conclusions

In this study we found history of HDP to be associated with midlife T2D development in parous women initially free of severe cardiometabolic disease, even when adjusting for established predictors of cardiometabolic deterioration. This suggests that history of HDP as a clinical predictor of T2D development in middle aged women warrants further study. Women affected by HDP also had worse cardiovascular risk factor status at age 50 years, which roughly corresponded to 10 years of older age. In conclusion, our results suggest that information on previous HDP is not only relevant for the risk of cardiometabolic disease during the decades post-pregnancy, but might also be clinically useful to predict middle age T2D development above and beyond established risk factors.

## Additional file

**Additional file 1:** Additional methods and results. **Table S1**: Descriptive comparison of cardiometabolic status at age 50 years between women alive at age 60 years with or without clinical visit. **Table S2**: Descriptive comparison of cardiometabolic status between women with and without full reproductive history at age 50 years.

## Abbreviations

BMI: body mass index
CVD: cardiovascular disease
DBP: diastolic blood pressure
GDM: gestational diabetes mellitus
HDP: hypertensive disorders of pregnancy
OGTT: oral glucose tolerance test
OR: odds ratio
RR: relative risk
SBP: systolic blood pressure
T2D: type 2 diabetes
