# Supplementary material for: Midlife development of type 2 diabetes and hypertension in women by history of hypertensive disorders of pregnancy
Source: Cardiovasc Diabetol. 2018 Sep 10;17:124. doi: 10.1186/s12933-018-0764-2 (PMC6130069; doi:10.1186/s12933-018-0764-2)
Supplement: Supplementary file 1 — Additional file 1: Additional methods and results. Table S1: Descriptive comparison of cardiometabolic status at age 50 years between women alive at age 60 years with or without clinical visit. Table S2: Descriptive comparison of cardiometabolic status between women with and without full reproductive history at age 50 years. [file 12933_2018_764_MOESM1_ESM.docx]

**ADDITIONAL FILE 1**

**Cardiovascular Diabetology**

**Midlife development of type 2 diabetes and hypertension in women by history of hypertensive disorders of pregnancy**

Timpka et al.

**Additional Methods**

International classification of disease code definitions

*Hypertensive disorders of pregnancy*

ICD-8: 637.01, 637.03, 637.04, 637.09, 637.10, 661.2; ICD-9: 642A, 642D, 642E, 642F, 642G, 642X; ICD-10: O13, O14, O15

*Diabetes mellitus or gestational diabetes mellitus*

ICD-8: 250; ICD-9: 250, 6480, 648A; ICD-10: O24, E10, E11

Acquiring the local delivery registry data

The data in the local birth registry had originally been lost. However, by matching data in current registries with data in a register file copy without unique identifiers, Statistics Sweden managed to uniquely identify the delivering woman in 99.2% for all deliveries in the local registry 1955-1972.

**Additional Results**

| **Table S1** – Descriptive comparison of cardiometabolic status at age 50 years between women alive at age 60 years with or without clinical visit | | | | |
| --- | --- | --- | --- | --- |
|  | Clinical visit also at age 60±1 years (N=6,641) | | No clinical visit at age 60±1 years (N=1,514) | |
|  |  | Missing, N |  | Missing, N |
| Age, years Mean±SD | 50.1±0.3 | – | 50.1±0.3 | – |
| History of HDP, N (%) | 289 (4.4) | – | 58 (3.8) | – |
| Family history of CVD, N (%)^*^ | 1,899 (28.6) | 1 | 360 (23.8) | 31 |
| Family history of diabetes, N (%)^*^ | 2,080 (31.3) | 2 | 351 (23.2) | 26 |
| Hypertension, N (%) | 1,580 (23.8) | 86 | 426 (28.1) | 32 |
| MAP, mmHg Mean±SD | 96±13 | 102 | 97±14 | 36 |
| SBP, mmHg Mean±SD | 128±19 | 95 | 130±21 | 35 |
| DBP, mmHg Mean±SD | 80±12 | 101 | 81±12 | 36 |
| BMI, kg/m^2^ Mean±SD | 25.4±4.0 | 51 | 25.9±4.4 | 21 |
| 2h glucose post OGTT, mmol/l Mean±SD | 6.79±1.3 | 279 | 6.93±1.5 | 65 |
| Education level, N (%) |  | 1 |  | 2 |
| - Elementary school or less | 1,242 (18.7) |  | 313 (20.7) |  |
| - High School | 3,569 (53.7) |  | 753 (49.7) |  |
| - College/University | 1,829 (27.5) |  | 446 (29.5) |  |
| Cholesterol, mmol/l Median (IQR) | 5.63 (5.00; 6.36) | 91 | 5.73 (5.08 ; 6.50) | 28 |
| Smoking, N (%) | 1,517 (22.8) | – | 474 (31.3) | – |
| BMI: Body mass index; CVD: Cardiovascular disease; DBP: Diastolic blood pressure; HDP: Hypertensive disorders of pregnancy; MAP: Mean arterial blood pressure; OGTT: Oral glucose tolerance test; SBP: Systolic blood pressure; SD: Standard deviation  * Variable combines answers at both age 50 and age 60 years visits.  Percentages are not always adding up to 100% due to rounding.  Dashes in columns are markers of no missing data in the study sample. | | | | |

| **Table S2** – Descriptive comparison of cardiometabolic status between women with and without full reproductive history at age 50 years | | |
| --- | --- | --- |
| **Variable** | **Women with full reproductive history**  (N=8,720) | **Women without full reproductive history**  (N=2,524) |
| Age, years Mean±SD | 50.1±0.3 | 50.1±0.2 |
| Education level, N (%) |  |  |
| - Elementary school or less | 1,691 (19.4) | 271 (10.7) |
| - High School | 4,644 (53.3) | 1,063 (42.1) |
| - College/University | 2,382 (27.3) | 1,190 (47.2) |
| - Missing | 3 (0.03) | – |
| Family history of CVD, N (%) |  |  |
| - Yes | 2,439 (28.0) | 654 (25.9) |
| - No | 6,241 (71.6) | 1,864 (73.9) |
| - Missing | 40 (0.5) | 6 (0.2) |
| Family history of diabetes, N (%) |  |  |
| - Yes | 2,669 (30.6) | 738 (29.2) |
| - No | 6,017 (69.0) | 1,778 (70.4) |
| - Missing | 34 (0.4) | 8 (0.3) |
| Smoking, N (%) |  |  |
| - Yes | 2,145 (24.6) | 581 (23.0) |
| - No | 6,575 (75.4) | 1,943 (77.0) |
| Diabetes, N (%) |  |  |
| - Yes | 229 (2.6) | 72 (2.9) |
| - No | 8,491 (97.4) | 2,451 (97.1) |
| - Missing | – | 1 (0.04) |
| Hypertension, N (%) |  |  |
| - Yes | 2,237 (25.7) | 518 (20.5) |
| - No | 6,361 (73.0) | 1,982 (78.5) |
| - Missing | 122 (1.4) | 24 (1.0) |
| SBP, mmHg Mean±SD | 129±19 | 125±18 |
| DBP, mmHg Mean±SD | 80±12 | 78±11 |
| BMI, kg/m^2^ Mean±SD | 25.6±4.2 | 25.2±4.0 |
| Cholesterol, mmol/l Median (IQR) | 5.66 (5.00; 6.40) | 5.59 (4.95; 6.26) |
| 2h glucose post OGTT, mmol/l Mean±SD | 6.89±1.5 | 6.85±1.5 |

BMI: Body mass index; CVD: Cardiovascular disease; DBP: Diastolic blood pressure; HDP: Hypertensive disorders of pregnancy; OGTT: Oral glucose tolerance test; SBP: Systolic blood pressure; SD: Standard deviation

Percentages are not always adding up to 100% due to rounding.

Dashes in columns are markers of no missing data in the study sample.
